# *ABCG2* Contributes to Multidrug Resistance and Aggressive Phenotypes Associated with ERK Signaling in Gastric Cancer

**DOI:** 10.3390/ijms27115039

**Published:** 2026-06-02

**Authors:** Özlem Türksoy Terzioğlu, Gökhan Terzioğlu

**Affiliations:** 1 Department of Molecular Biology and Genetics, Hamidiye Institute of Health Sciences, University of Health Sciences, Istanbul 34899, Türkiye; ozlem.turksoy@sbu.edu.tr; 2 Experimental Medicine Research and Application Center (DETUAM), Validebag Research Park, University of Health Sciences, Istanbul 34662, Türkiye; 3 Department of Medical Biology, Hamidiye International School of Medicine, University of Health Sciences, Istanbul 34899, Türkiye

**Keywords:** *ABCG2*, ERK signaling, gastric cancer, multidrug resistance, stemness, epithelial–mesenchymal transition, paclitaxel resistance

## Abstract

Multidrug resistance remains a major obstacle in gastric cancer therapy and is frequently associated with aggressive phenotypes. Although *ABCG2* is a well-known drug efflux transporter, its functional contribution to paclitaxel (PTX) resistance and its relationship with ERK signaling in gastric cancer remain incompletely understood. In this study, PTX-resistant gastric cancer cell models were established through prolonged drug exposure. Resistant cells exhibited cross-resistance to cisplatin and 5-fluorouracil together with enhanced drug efflux activity, invasion capacity, spheroid formation, stemness-associated marker expression, and G0/G1 enrichment. *ABCG2* expression was markedly increased in resistant cells. Stable knockdown of *ABCG2* restored PTX sensitivity and significantly reduced drug efflux, invasion, spheroid formation, and stemness-associated phenotypes, while increasing apoptosis and altering cell cycle distribution. *ABCG2* depletion was associated with reduced ERK phosphorylation and decreased expression of ERK downstream target genes. Pharmacological inhibition of ERK signaling similarly suppressed resistance-associated phenotypes and reduced *ABCG2* expression. Whereas reactivation of ERK signaling by constitutively active MEK1 partially rescued the effects of *ABCG2* depletion, restoring aggressive and multidrug-resistant phenotypes. Our findings indicate that ERK signaling functionally contributes to *ABCG2*-associated multidrug resistance and aggressive phenotypes in PTX-resistant gastric cancer cells.

## 1. Introduction

Gastric cancer (GC) remains a substantial public health concern globally, being the fifth most common cancer and the fourth leading cause of cancer-associated death, with nearly 1.1 million incident cases and 770,000 fatalities reported in 2020 [1]. Despite advances in systemic therapy, paclitaxel (PTX)-based chemotherapy remains ineffective in treating advanced gastric cancer because of the rapid development of multidrug resistance (MDR). These limitations highlight the urgency of developing strategies to improve the effectiveness of current chemotherapy drugs and to enhance survival rates in patients with gastric cancer [2].

MDR in cancer cells is often associated with alterations in epithelial–mesenchymal transition (EMT), cancer stem cell (CSC) plasticity, and cell cycle dynamics. All of them help tumor cells to evade cytotoxic suppression and relapse after treatment. A key molecular determinant of MDR, ATP-binding cassette subfamily G member 2 (*ABCG2*), is a member of the ATP-binding cassette (ABC) transporter family. It was first characterized in doxorubicin-resistant MCF-7 breast cancer cells and linked to the MDR phenotype [3,4]. Its overexpression and functional role in chemoresistance have been reported in multiple tumor types [5,6,7]. *ABCG2* is increasingly recognized as a CSC-associated marker across many malignancies. It contributes to key processes such as tumor initiation, metastatic progression, recurrence, and drug resistance, highlighting its therapeutic relevance [8,9,10]. *ABCG2* expression has also been reported to be enriched in poorly differentiated gastric tumors and gastric cancer stem-like cell populations. This suggests that *ABCG2*-associated resistance mechanisms may be especially relevant in this malignancy [10,11,12,13]. Gastric cancer, therefore, represents a clinically relevant model for investigating PTX resistance because of the frequent emergence of MDR during second-line therapy [14,15]. Although *ABCG2* has been associated with gastric cancer, its role in PTX-induced MDR and its relationship with CSC-associated phenotypes and EMT programs are not fully understood. The signaling pathways through which *ABCG2* may regulate these aggressive phenotypes have not yet been fully elucidated. Emerging evidence suggests that ERK signaling plays an important role in drug resistance and CSC maintenance [16,17,18]. However, the functional relationship between *ABCG2* expression and ERK signaling in PTX-resistant gastric cancer cells remains unclear.

This study aimed to investigate the role of *ABCG2* in PTX-induced multidrug resistance and to determine whether ERK signaling contributes to *ABCG2*-associated aggressive phenotypes in gastric cancer cells. We hypothesized that *ABCG2* may contribute to chemoresistance-associated phenotypes linked to ERK signaling, CSC plasticity, and EMT. To test this hypothesis, we created PTX-resistant gastric cancer cell models and examined the effects of *ABCG2* silencing, ERK inhibition, and ERK reactivation on drug sensitivity, stemness-associated features, invasion capacity, and efflux activity.

## 2. Results

### 2.1. Drug-Resistant Gastric Cancer Cells Can Develop a Multidrug-Resistant Phenotype

**Figure 1:**
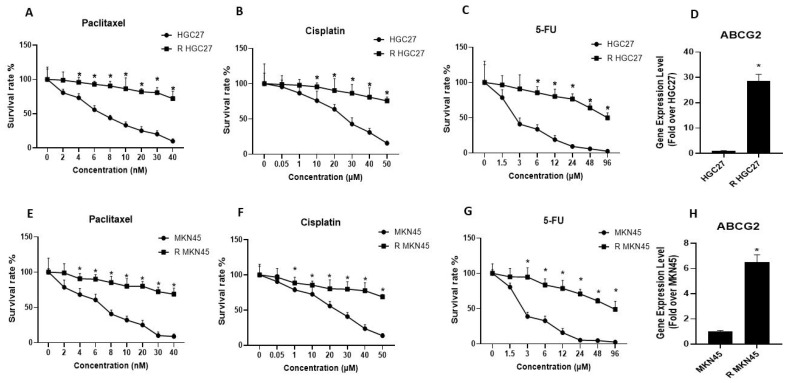
Chemotherapeutic drug response and *ABCG2* expression in parental and resistant gastric cancer cells. (**A**–**C**) Cell survival rates of parental HGC27 and resistant R HGC27 cells following treatment with increasing concentrations of PTX (**A**), cisplatin (**B**), and 5-FU (**C**), as determined by MTT assay. (**D**) Relative *ABCG2* mRNA expression levels in HGC27 and R HGC27 cells. (**E**–**G**) Cell survival rates of parental MKN45 and resistant R MKN45 cells treated with PTX, (**E**), cisplatin, (**F**), and 5-FU (**G**). (**H**) Relative *ABCG2* expression levels in MKN45 and R MKN45 cells. Data are presented as mean ± SD. * *p* < 0.05 indicates a statistically significant difference from the corresponding parental control cells.

In this study, human gastric cancer cell lines HGC27 and MKN45 were used. PTX-resistant HGC27 cells (R HGC27) and MKN45 (R MKN45) were generated by stepwise adaptation of parental cells to increasing concentrations of PTX. It was initiated at one-fiftieth of the IC_50_ value, with 25% dose increments applied every two weeks. This selection process resulted in a cell population capable of sustained growth, even at PTX concentrations as high as 12.81 nM for HGC27 and 11.9 nM for MKN45. The IC_50_ of PTX was 12.81 ± 0.8 nM in HGC27 parental cells, whereas R HGC27 cells exhibited a markedly elevated IC_50_ of 72.88 ± 5.6 nM (Figure 1A). In addition, R HGC27 cells demonstrated cross-resistance to multiple chemotherapeutic agents with distinct mechanisms of action (Cisplatin IC50:27.57 µM for parental, 107.11 µM for R HGC27, and 5-FU IC50:4.35 µM for parental, 80.09 µM for R HGC27) (Figure 1B,C). Accordingly, this cell population was designated as multidrug-resistant HGC27 cells (R HGC27).

The IC_50_ value of PTX in parental MKN45 cells was 11.9 ± 1.1 nM, whereas PTX-resistant MKN45 cells showed a substantially increased IC_50_ of 61.46 ± 6.2 nM (Figure 1E). PTX-resistant MKN45 cells displayed significant cross-resistance to chemotherapeutic agents with different mechanisms of action, including cisplatin (IC_50_: 24.83 µM in parental cells vs. 89.64 µM in R MKN45 cells) and 5-FU (IC_50_: 2.64 µM in parental cells vs. 81.92 µM in R MKN45 cells) (Figure 1F,G).

qRT-PCR analysis revealed a significant upregulation of *ABCG2* expression in resistant cells compared to their parental counterparts. This indicates an association between *ABCG2* overexpression and the multidrug-resistant phenotype (*p* < 0.05) (Figure 1D,H).

### 2.2. Evaluation of ABCG2 Expression and Functional Effects Following ABCG2 Knockdown

**Figure 2:**
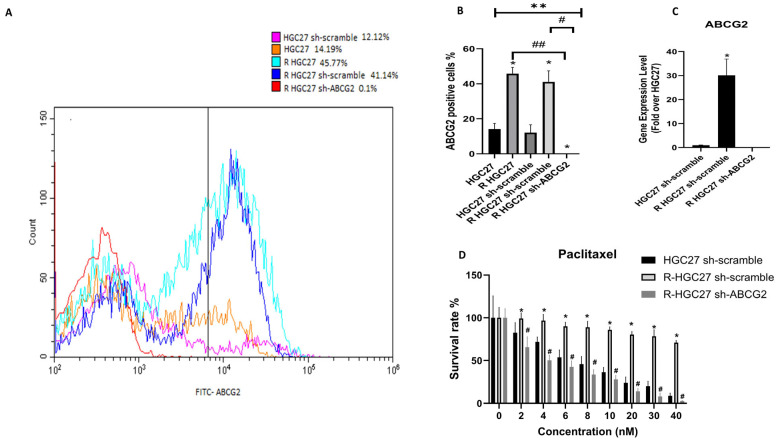
*ABCG2* expression contributes to paclitaxel resistance in HGC27 gastric cancer cells. (**A**) Representative flow cytometry histograms showing the percentage of *ABCG2*-positive cells in the following groups: parental HGC27 cells, paclitaxel-resistant HGC27 (R HGC27) cells, and *ABCG2* knockdown cells. (**B**) Quantification of the percentage of *ABCG2*-positive cells measured by flow cytometry in the experimental groups. (**C**) qRT-PCR analysis showing relative *ABCG2* mRNA expression levels and confirming efficient knockdown of *ABCG2* in resistant HGC27 cells. (**D**) Paclitaxel chemoresistance assay demonstrating that *ABCG2* knockdown significantly reduces cell survival in R HGC27 cells compared to scramble controls, indicating the restoration of drug sensitivity. * *p* < 0.05 indicates comparison between HGC27 sh-scramble cells and other groups; # *p* < 0.05 indicates comparison between R H GC27 sh-scramble cells and R HGC27 sh-*ABCG2* cells. ** *p* < 0.05 indicates comparison between HGC27 cells and R HGC27 sh-*ABCG2* cells. ## *p* < 0.05 indicates comparison between R HGC27 and R HGC27 sh-*ABCG2* cells. Data are presented as mean ± SD.

As comparable multidrug resistance and *ABCG2* upregulation were observed in both HGC27 and MKN45 cells, HGC27 cells were selected for subsequent mechanistic analysis. *ABCG2* expression and the number of *ABCG2*-positive cells were higher in the multidrug-resistant cells. To evaluate the functional consequences of *ABCG2* knockdown in multidrug-resistant gastric cancer cells, *ABCG2* expression, drug sensitivity, apoptosis, and cell cycle distribution were analyzed in control and multidrug-resistant HGC27 cells. Flow cytometry analysis revealed a marked increase in *ABCG2* expression in R HGC27 sh-scramble cells compared with HGC27 sh-scramble cells, whereas *ABCG2* knockdown significantly reduced the proportion of *ABCG2*-positive cells and *ABCG2* gene expression (Figure 2A–C).

*ABCG2* silencing significantly increased the sensitivity of resistant HGC27 cells to PTX (IC50 7.06 ± 0.06 nM), as demonstrated by a pronounced decrease in cell survival with increasing drug concentrations (Figure 2D). These findings indicate that *ABCG2* contributes to PTX resistance in HGC27 cells.

**Figure 3:**
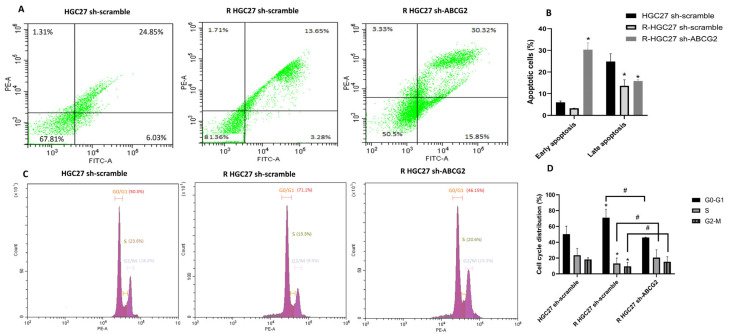
*ABCG2* knockdown is associated with increased apoptosis and altered cell cycle distribution in PTX-resistant HGC27 cells. (**A**) Representative Annexin V–FITC/PI flow cytometry plots showing the apoptotic cell population. (**B**) Quantification of early and late apoptotic cells. (**C**) Representative cell cycle distribution profiles analyzed using flow cytometry. (**D**) Quantification of cell cycle distribution in G0/G1, S, and G2/M phases. * *p* < 0.05 indicates comparison between HGC27 sh-scramble cells and other groups; # *p* < 0.05 indicates comparison between R HGC27 sh-scramble cells and R HGC27 sh-*ABCG2* cells. Data are presented as mean ± SD.

Furthermore, Annexin V/PI staining showed that PTX treatment at the IC_50_ concentration for each group induced significantly higher levels of apoptosis in R HGC27 sh-*ABCG2* cells than in the other cell groups (Figure 3A,B). *ABCG2* knockdown significantly increased both early and late apoptotic cell populations in resistant HGC27 cells following PTX treatment (Figure 3A,B).

Many chemotherapeutic agents show cytotoxic activity by selectively targeting proliferating cells. However, such strategies are often ineffective against stemness-associated phenotypes (CSCs), which are typically found in a quiescent G0 state. The elimination of quiescent CSC populations remains a major therapeutic challenge [19]. Similarly, cell cycle analysis revealed significant alterations in phase distribution between drug-resistant and parental HGC27 cells (Figure 3C,D). Compared with parental cells, R HGC27 cells exhibited an increased proportion in the G0/G1 phase and a reduced proportion in the S and G2/M phases. This was associated with an impaired apoptotic responsiveness to PTX. *ABCG2* knockdown decreased the proportion of cells in the G0/G1 phase and increased the number of cells in the S and G2/M phases compared with R HGC27 cells (Figure 3C,D).

These results show that *ABCG2* contributes to PTX resistance in HGC27 cells by decreasing drug-induced apoptosis and stimulating G0/G1 accumulation. Targeting *ABCG2* restores PTX sensitivity in R HGC27 cells.

### 2.3. ABCG2 Depletion Suppresses Invasion, Stemness-Associated Features, and Drug Efflux in R HGC27 Cells

**Figure 4:**
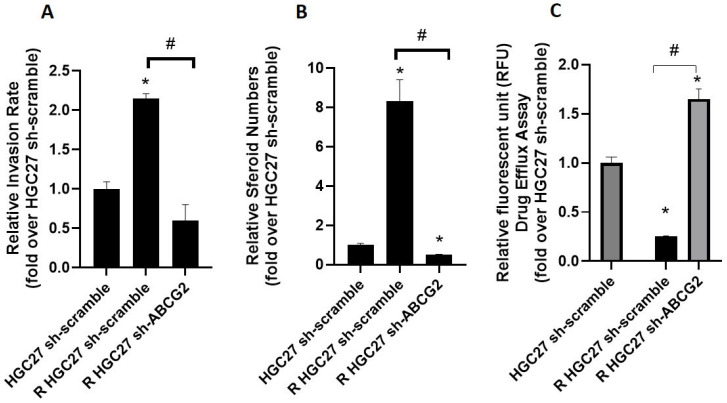
*ABCG2* knockdown suppresses the invasive and stem-like properties, as well as efflux activity, in resistant HGC27 cells. (**A**) Quantification of the invasion rate of HGC27 sh-scramble, R HGC27 sh-scramble, and R HGC27 sh-*ABCG2* cells. (**B**) Spheroid formation assay showing the number of spheroids. (**C**) Efflux pump activity measured by fluorescence-based assay. R HGC27 sh-scramble cells exhibit increased invasion, spheroid formation, and efflux activity, all of which are significantly reduced by *ABCG2* knockdown. Data are presented as mean ± SD. * *p* < 0.05 indicates comparison between HGC27 sh-scramble cells and other groups; # *p* < 0.05 indicates comparison between R HGC27 sh-scramble cells and R HGC27 sh-*ABCG2* cells.

To investigate the role of *ABCG2* in the aggressive phenotype of resistant gastric cancer cells, the invasion capacity, spheroid-forming ability, and efflux pump activity of parental and resistant HGC27 cells were assessed. Transwell invasion assays revealed that R HGC27 sh-scramble cells showed a significantly higher invasion rate than HGC27 sh-scramble cells. Silencing *ABCG2* in resistant cells reduced their invasive capacity (Figure 4A). Consistently, spheroid formation assays showed a significant increase in spheroid numbers in R HGC27 sh-scramble cells, indicating enhanced stem-like properties associated with drug resistance. In contrast, *ABCG2* knockdown significantly suppressed spheroid formation in resistant cells (Figure 4B). To further evaluate the functional capacity associated with stemness, a limiting dilution sphere formation assay was performed under single-cell seeding conditions. R HGC27 cells exhibited a higher frequency of spheroid-positive wells than parental cells, indicating an increased capacity for sphere initiation. Silencing of *ABCG2* significantly decreased the capacity of sphere initiation (Supplementary Figure S8). Additionally, analysis of efflux pump activity revealed significantly reduced intracellular fluorescence in R HGC27 sh-scramble cells, indicating an increased capacity for drug efflux. Silencing *ABCG2* significantly decreased efflux pump activity (Figure 4C). These results suggest that *ABCG2* is related to increased capacity of invasion, spheroid formation, and efflux pump activity in R HGC27 cells. Thus, targeting *ABCG2* can effectively decrease these aggressive and drug-resistant characteristics.

### 2.4. ABCG2 Knockdown Modulates Stemness and EMT-Related Gene Expression in Multidrug-Resistant HGC27 Cells

**Figure 5:**
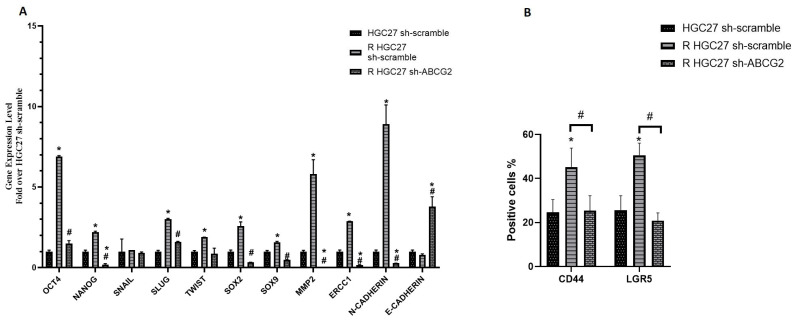
*ABCG2* knockdown significantly alters stemness and EMT-related gene expression and decreases the CD44- and LGR5-positive cell populations in multidrug-resistant HGC27 cells. (**A**) Relative mRNA expression levels are analyzed by quantitative real-time PCR in HGC27 sh-scramble, resistant HGC27 (R HGC27) sh-scramble, and R HGC27 sh-*ABCG2* cells. (**B**) Quantification of the percentage of CD44- and LGR5-positive cells determined by flow cytometry analysis. * *p* < 0.05 indicates comparison between HGC27 sh-scramble cells and other groups; # *p* < 0.05 indicates comparison between R HGC27 sh-scramble cells and R HGC27 sh-*ABCG2* cells. Data are presented as mean ± SD.

*ABCG2* knockdown markedly affected the expression of stemness- and EMT-related genes in multidrug-resistant HGC27 cells (Figure 5A). Compared with parental HGC27 sh-scramble cells, R HGC27 sh-scramble cells exhibited significantly elevated mRNA levels of several stemness markers, including *OCT4*, *NANOG*, *SLUG*, *TWIST*, *SOX2*, and *SOX9* (* *p* < 0.05). In addition, EMT-associated genes such as *SNAIL*, *MMP2*, *ERCC1*, and *N-CADHERIN* were upregulated in resistant cells, suggesting a more aggressive stem-like phenotype.

Silencing of *ABCG2* in resistant HGC27 cells (R HGC27 sh-*ABCG2*) significantly reduced the expression levels of stemness- and EMT-related genes compared with those in R HGC27 sh-scramble cells (# *p* < 0.05). In contrast, the expression of the epithelial marker *E-CADHERIN* was increased following *ABCG2* knockdown.

These findings demonstrate that *ABCG2* contributes to the maintenance of stemness and EMT-related gene expression in multidrug-resistant HGC27 cells. This suggests a potential role for *ABCG2* in regulating aggressive phenotypic characteristics associated with chemoresistance.

### 2.5. ABCG2 Silencing Decreases CD44- and LGR5-Positive Cell Populations in Resistant Cells

To investigate whether *ABCG2* contributes to the maintenance of stem-like cell populations in chemoresistant gastric cancer cells, the proportions of CD44- and LGR5-positive cells were assessed using flow cytometry. R HGC27 sh-scramble cells exhibited a significantly greater proportion of CD44- and LGR5-positive cells than the parental HGC27 sh-scramble population (Figure 5B and Supplementary Figure S10).

Silencing *ABCG2* in R HGC27 cells resulted in a marked decrease in CD44- and LGR5-positive cell populations. These findings demonstrate that *ABCG2* plays a critical role in maintaining stem-like characteristics in R HGC27 cells. *ABCG2* knockdown effectively attenuates the expression of stemness-associated markers.

### 2.6. ERK Signaling Modulates ABCG2 Expression, Efflux Function, and PTX Resistance in Resistant Cells

**Figure 6:**
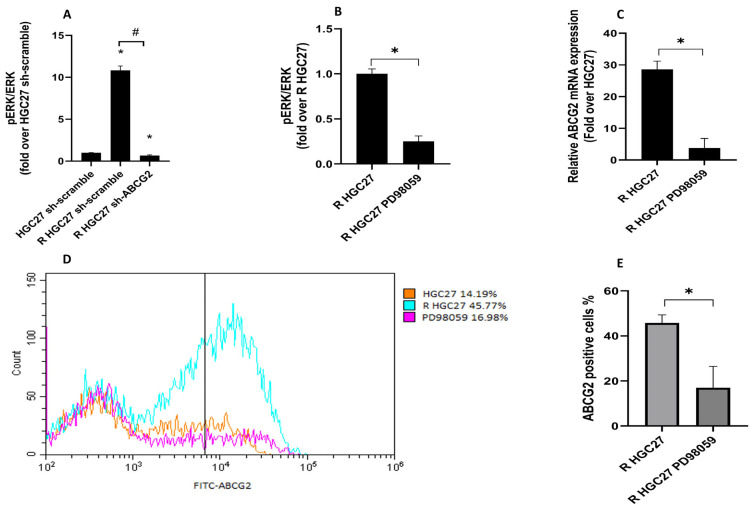
ERK signaling regulates *ABCG2* expression in R HGC27 cells. (**A**) ERK activation status is assessed by pERK/ERK ratio in HGC27 sh-scramble, R HGC27, and R HGC27 sh-*ABCG2* cells. *ABCG2* silencing markedly reduces ERK phosphorylation in the resistant cells. (**B**) Pharmacological inhibition of ERK signaling with PD98059 significantly decreases pERK/ERK levels in R HGC27 cells. (**C**) Relative *ABCG2* mRNA expression in R HGC27 cells treated with PD98059 compared to that in untreated R HGC27 cells. (**D**) Representative flow cytometry histograms showing *ABCG2* expression in HGC27, R HGC27, and PD98059-treated R HGC27 cells. (**E**) Quantification of *ABCG2*-positive cell population. Data are presented as mean ± SD. * *p* < 0.05; # *p* < 0.05 vs. R HGC27 sh-scramble.

To investigate the involvement of ERK signaling in *ABCG2*-related drug resistance, we analyzed the ERK activation and *ABCG2* expression in R HGC27 cells. The pERK/ERK ratio was significantly higher in R HGC27 sh-scramble cells than in parental counterparts. In contrast, *ABCG2* knockdown markedly decreased ERK activation (Figure 6A). Immunofluorescence analysis revealed increased nuclear accumulation of pERK in resistant cells. This accumulation was decreased after silencing *ABCG2*. These findings suggest a functional association between *ABCG2* expression and ERK signaling activity (Supplementary Figure S9).

Also, pharmacological inhibition of ERK signaling using PD98059 significantly decreased the pERK/ERK ratio in the R HGC27 cells (Figure 6B). PD98059 treatment significantly suppressed *ABCG2* mRNA expression. It reduced *ABCG2* protein levels, as demonstrated by flow cytometry analysis (Figure 6C–E).

**Figure 7:**
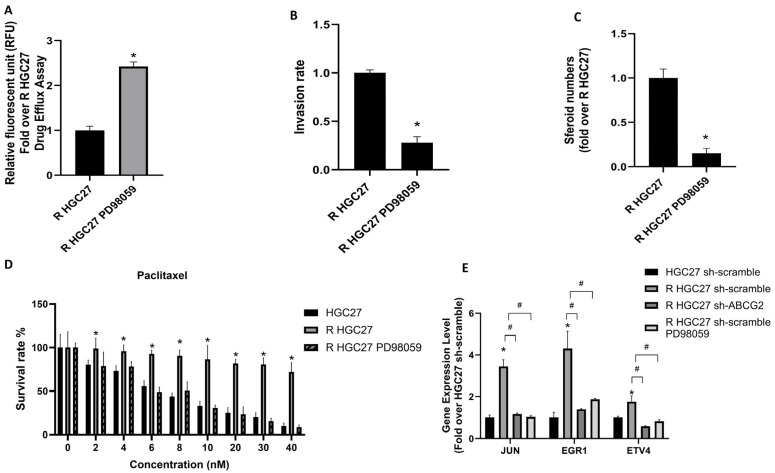
ERK signaling regulates aggressive phenotypes in R HGC27 cells. (**A**) Drug efflux activity is expressed in relative fluorescence units (RFUs). (**B**) Invasion capacity of R HGC27 cells with or without ERK inhibition. (**C**) Spheroid-forming ability of R HGC27 cells following PD98059 treatment. (**D**) Cell survival rates following PTX treatment in HGC27, R HGC27, and PD98059-treated R HGC27 cells. (**E**) qRT-PCR analysis of relative mRNA expression levels of *JUN*, *EGR1*, and *ETV4* in parental HGC27 sh-scramble, R HGC27 sh-scramble, R HGC27 sh-*ABCG2*, R HGC27 sh-scramble + PD98059. The expression levels are normalized to those of HGC27 sh-scramble cells. Data are represented as the mean ± SD. * *p* < 0.05 vs. HGC27 sh-scramble; # *p* < 0.05 vs. R HGC27 sh-scramble.

In parallel, suppression of ERK pathway activity significantly increased intracellular fluorescence during drug efflux analysis, indicating reduced efflux pump activity (Figure 7A). In addition, the sphere-forming and invasion capacities of R HGC27 cells decreased (Figure 7B,C).

Functional analysis showed that inhibiting ERK significantly increased the sensitivity of R HGC27 cells to PTX, as demonstrated by decreased cell survival at increasing drug concentrations (Figure 7D). Limiting dilution assay also showed a decreased sphere initiation capacity upon ERK inhibition (Supplementary Figure S8).

Increased ERK activation in resistant cells was accompanied by elevated expression of the downstream ERK target genes *JUN*, *EGR1*, and *ETV4*. Knockdown of *ABCG2* and pharmacological inhibition of ERK signaling with PD98059 significantly reduced *ABCG2* expression levels (Figure 7E).

These findings suggest a functional link between ERK signaling and *ABCG2*-mediated efflux activity, which could contribute to multidrug resistance in HGC27 cells. Targeting ERK-related *ABCG2* activity could therefore help to reduce the resistant phenotype.

### 2.7. ERK Signaling Restores Chemoresistance and Aggressive Phenotypes in ABCG2-Depleted R HGC27 Cells

**Figure 8:**
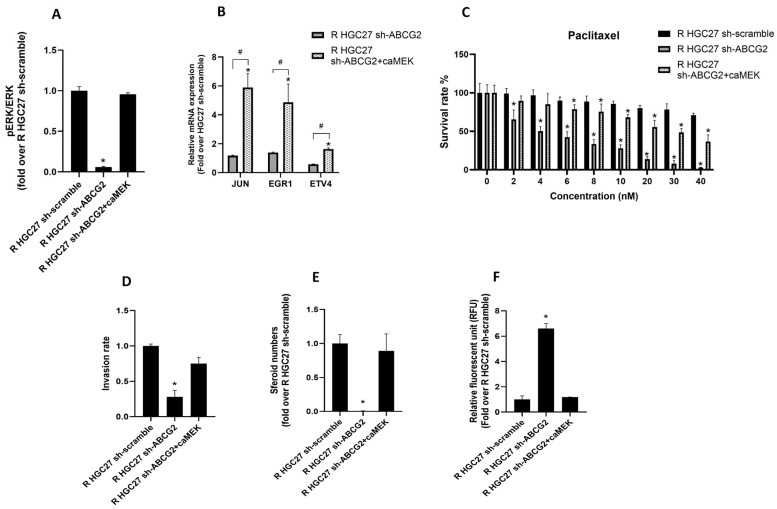
Reactivation of ERK signaling by constitutively active MEK1 rescues the aggressive phenotype suppressed by *ABCG2* knockdown in R HGC27 cells. (**A**) ERK activation is evaluated using the pERK/ERK ratio in R HGC27 sh-scramble, sh-*ABCG2*, or sh-*ABCG2* cells with constitutively active MEK1 (caMEK1). *ABCG2* silencing markedly reduces ERK phosphorylation, a reduction that is restored by caMEK1 expression. (**B**) qRT-PCR analysis of relative mRNA expression levels of *JUN*, *EGR1*, and *ETV4* in R HGC27 sh-*ABCG2*, and rescue cells (R HGC27 sh-*ABCG2* + caMEK1). (**C**) Cell survival rates following PTX treatment in R HGC27 sh-scramble, R HGC27 sh-*ABCG2*, and R HGC27 sh-*ABCG2* + caMEK1 cells. caMEK1 expression significantly reverses the chemosensitive phenotype induced by *ABCG2* knockdown. (**D**) Invasion capacity, (**E**) spheroid formation ability, and (**F**) drug efflux activity of R HGC27 cells following *ABCG2* depletion and caMEK1 reconstitution. Data are shown as mean ± SD. * *p* < 0.05 compared with R HGC27 sh-scramble; # *p* < 0.05 vs. R HGC27 sh-*ABCG2*.

To functionally confirm the involvement of ERK signaling, constitutively active MEK1 (caMEK1), the upstream activator of ERK phosphorylation, was ectopically expressed in *ABCG2*-knockdown R HGC27 cells to restore the attenuated ERK activity (Figure 8A). Restoring ERK activity in R HGC27 sh-*ABCG2* cells increased the expression levels of the ERK downstream target genes, *JUN*, *EGR1*, and *ETV4*. These findings further support a functional role for ERK signaling in mediating *ABCG2*-associated multidrug resistance phenotypes (Figure 8B). Reconstitution of ERK signaling by caMEK1 markedly restored PTX resistance in *ABCG2*-depleted R HGC27 cells (Figure 8C) and restored sphere formation, invasion and drug efflux capacity (Figure 8D). Restoration of ERK activity by constitutively active MEK partially rescued sphere initiation capacity in *ABCG2*-silenced cells, supporting the contribution of ERK signaling to stemness-associated phenotypes in multidrug-resistant gastric cancer cells (Supplementary Figure S8).

Kaplan–Meier survival analyses demonstrated that elevated *ABCG2* expression was significantly associated with poorer overall survival in gastric cancer patients in both the KM-plotter and TCGA-STAD cohorts (Supplementary Figure S1). Similarly, increased expression of MAPK1 and MAPK3 was associated with decreased overall survival, supporting the clinical relevance of ERK pathway activation in gastric cancer (Supplementary Figure S2). Analysis of the TCGA-STAD cohort using the UCSC Xena browser revealed significant positive correlations between *ABCG2* expression and ERK pathway components (*MAPK3, JUN, FOS*, and *EGR1*), and stemness-associated markers (*LGR5* and *NANOG*) (Supplementary Figure S3). Similar correlation patterns were observed in the cBioPortal analysis of the TCGA-STAD cohort. This further supports the association between *ABCG2* expression and ERK signaling, as well as stemness-related transcriptional programs (Supplementary Figure S4).

UALCAN/CPTAC proteomic analysis revealed variations in the expression levels of MAPK1 and MAPK3 proteins across different gastric cancer subtypes. Expression levels of MAPK1 and MAPK3 were markedly decreased in the S4 subtype, whereas relatively higher levels were observed in the S6 and S7 subtypes. Significant differences were detected in several comparisons between subtypes, particularly between the S2–S4 and S3–S6 groups. These findings further suggest heterogeneous activity of the ERK pathway among gastric cancer subtypes (Supplementary Figure S5).

RPPA-based co-expression analysis of TCGA-STAD samples revealed a modest yet significant positive correlation between *ABCG2* mRNA expression and phosphorylated *JUN* (*JUN*_pS73), as well as between *ABCG2* expression and phosphorylated RPS6KA1 (RPS6KA1_pT359/S363). This supports the potential association between *ABCG2* expression and ERK-related signaling activity in gastric cancer (Supplementary Figure S6). Also, exploratory KEGG pathway enrichment analysis based on recurrently overexpressed genes identified in *ABCG2*-high TCGA-STAD gastric cancer samples demonstrated enrichment of oncogenic pathways associated with MAPK, PI3K-AKT, and ERBB signaling (Supplementary Figure S7 and Table S1).

These findings suggest that ERK signaling functionally contributes to *ABCG2*-associated MDR and aggressive phenotypes in gastric cancer cells.

## 3. Discussion

In this study, we analyzed the relationship between multidrug resistance and the aggressiveness-related characteristics in gastric cancer cells. PTX-resistant HGC27 cells showed cross-resistance to cisplatin and fluorouracil, and also exhibited increased drug efflux capacity, migratory behavior, and sphere-forming ability. These findings are consistent with multidrug resistance and stem cell-like phenotypes [20,21,22]. *ABCG2* expression was increased in multidrug-resistant cells. As qRT-PCR analysis showed, stemness-related markers, including *OCT4*, *NANOG*, *SOX2*, and *SOX9*, and mesenchymal markers, including *SLUG*, *TWIST*, and *N-CADHERIN*, were also increased, while the epithelial marker *E-CADHERIN* was decreased. These findings suggest that PTX-resistant gastric cancer cells exhibit stem cell-like and mesenchymal-like characteristics [20,21,22].

Cancer stemness is associated with chemoresistance, self-renewal, and cell mobility, which are associated with metastasis and recurrence [23]. The interplay among chemoresistance, *ABCG2* expression, and CSC- and EMT-associated properties, such as invasive behavior and spheroid-forming capacity, remains unclear. Therefore, the PTX-resistant HGC27 cell line, which exhibits multidrug resistance, was used for further investigation. Analysis of gene expression showed that the expression of stemness-related and mesenchymal genes decreased following *ABCG2* knockdown in multidrug-resistant cells. ATP-binding transporters, such as *ABCG2*, mediate drug efflux in cancer cells [24,25]. Cells expressing high levels of *ABCG2* have been shown to exhibit drug-resistant characteristics, CSC-, and EMT–like features [11,26]. Moreover, *ABCG2* has been suggested as a putative marker for cancer stem cells in gastric cancer [12]. We investigated the relationship between *ABCG2* expression and CSC-related traits, including drug efflux capacity, invasive potential, and spheroid-forming ability. Our findings revealed that *ABCG2* contributes to drug efflux, spheroid formation, and the migratory ability of drug-resistant cells, in line with earlier studies [20,27,28,29,30,31]. The expansion of the CD44^+^ and LGR5^+^ cell populations, together with enhanced spheroid-forming capacity and invasive potential, further supports the presence of stemness-associated and therapy-resistant phenotypes, as CD44 and LGR5 are well-established gastric CSC markers associated with self-renewal and therapy resistance [8,32]. These findings suggest that beyond its role in drug excretion, *ABCG2* contributes to multidrug resistance and aggressive phenotypes in PTX-resistant gastric cancer cells.

In our multidrug-resistant HGC27 cells, the G0/G1 phase cell ratio was significantly higher than in controls. Silencing the *ABCG2* gene reduced G0/G1 enrichment while significantly increasing apoptosis. These findings suggest that *ABCG2* contributes to the resistance phenotype not only by regulating drug elimination but also by modulating cell cycle distribution and survival signaling, which may reduce apoptotic responsiveness to PTX. ABC transporters, including *ABCG2*, are well-known mediators of decreased intracellular drug accumulation and increased chemoresistance [33]. Several reports describe how *ABCG2* suppression can lead to G0/G1 accumulation by modulating cyclins and CDK inhibitors (such as cyclin D3 and p21). The effects of *ABCG2* disruption on cell cycle phase distribution are context-dependent and may differ between cell types [34]. The increased apoptosis observed following *ABCG2* silencing is consistent with studies showing that *ABCG2* depletion or activity sensitizes cancer cells to cytotoxic agents and promotes apoptosis. This may occur either by increasing intracellular drug retention or by disrupting pro-survival signaling associated with the resistant state [33,35]. In summary, our data support the conclusion that *ABCG2* is associated with the maintenance of drug tolerance and survival in multidrug-resistant HGC27 cells. Targeting *ABCG2* shifts the cell cycle distribution towards the control profile and restores apoptotic responsiveness. These findings suggest that targeting *ABCG2* could help to increase PTX sensitivity in gastric cancer cells.

Extracellular signal-regulated kinase (ERK) is a key component of the mitogen-activated protein kinase (MAPK) signaling cascade. The components of this cascade are activated through tightly regulated, reversible phosphorylation events, thereby providing precise control of cell proliferation. ERK signaling plays a key role in regulating tumor cell survival, proliferation, and resistance to therapy across multiple cancer types [36,37,38]. Although *ABCG2* is primarily known as a membrane efflux transporter, ABC transporters may also indirectly affect intracellular signaling through cellular stress responses and receptor-associated signaling networks [33,39]. The precise molecular mechanism linking *ABCG2* activity to ERK activation requires further investigation.

In this study, *ABCG2* silencing significantly reduced ERK activation in PTX-resistant HGC27 cells, suggesting that ERK-related signaling may be associated with the resistant phenotype. In parallel, pharmacological inhibition of ERK signaling decreased *ABCG2* expression and significantly impaired spheroid formation, invasion capacity, and drug efflux activity in multidrug-resistant cells. Importantly, restoration of ERK activity by constitutively active MEK1 reversed the chemosensitive and stemness-suppressive effects induced by *ABCG2* depletion. Together, these findings support a functional contribution of ERK signaling to *ABCG2*-associated aggressive and stemness-related multidrug-resistant phenotypes. Previous evidence suggests that the MEK/ERK pathway may regulate *ABCG2* expression in a context-dependent manner. Although inhibiting the MEK/ERK/RSK axis has been associated with increased *ABCG2* expression, suppressing MEK/ERK signaling independently of RSK has been reported to decrease *ABCG2* levels [40]. Suppression of ERK signaling abolished the upregulation of Ribophorin II (RPN2)-mediated P-gp and *ABCG2* in SGC7901/DDP and SGC7901/VCR cells, suggesting that the ERK pathway plays a central role in RPN2-mediated multidrug resistance in gastric cancer [39]. Previous studies have linked ERK activation to drug resistance, EMT, and stemness-associated phenotypes. ERK signaling has been shown to regulate transcription factors associated with EMT drivers and to support survival under chemotherapy stress [21,38,41,42,43]. ERK activation was assessed using an ELISA-based phospho/total ERK approach rather than Western blotting. The functional role of ERK signaling was supported by additional pharmacological inhibition experiments and the rescue of the resistant phenotype by constitutively active MEK1. In addition, immunofluorescence analysis revealed increased nuclear accumulation of pERK in resistant cells, which was reduced following *ABCG2* knockdown. This observation provides further support for the idea that ERK activation is increased in resistant cells. Taken together, these findings suggest that ERK-related signaling may be related to *ABCG2*-associated resistance phenotypes. Consistently, several downstream transcriptional regulators of ERK have been linked to signaling programs associated with chemoresistance. This highlights the potential biological relevance of ERK pathway activation in multidrug-resistant cancer cells.

The decrease in the expression of stemness markers, EMT-associated phenotypes, and invasive behavior following *ABCG2* silencing suggests that *ABCG2* may contribute to multidrug resistance beyond its transporter function, potentially through ERK-related signaling programs associated with CSC plasticity. The data suggest that *ABCG2* expression and ERK-related signaling contribute to PTX resistance and aggressive phenotypes in HGC27 cells. Targeting this interaction may be a therapeutic approach for gastric cancer. In line with our experimental findings, survival analysis of publicly available datasets showed that high *ABCG2* expression was associated with worse overall survival in gastric cancer patients. Further survival analyses showed that elevated expression of MAPK1 and MAPK3 was significantly associated with poorer overall survival in patients with gastric cancer. This supports the clinical significance of ERK pathway activation. In addition, analyses of publicly available clinical datasets, including TCGA cohorts, revealed significant positive correlations between *ABCG2* expression and several ERK pathway-associated components, including MAPK3, *JUN*, and *EGR1* (Supplementary Figures S1–S4).

Proteomic analyses using CPTAC/UALCAN revealed subtype-dependent differences in MAPK1 and MAPK3 expression in gastric cancer cohorts (Supplementary Figure S5). This suggests that ERK-related signaling may vary across gastric cancer subtypes. In addition, analyses of TCGA-STAD RPPA datasets revealed a modest positive correlation between *ABCG2* expression and the phosphorylation of *JUN* and RPS6KA1. This finding supports the relationship between *ABCG2* expression and ERK-related signaling activity in gastric cancer [44,45,46] (Supplementary Figure S6). An exploratory pathway enrichment analysis based on the identification of recurrently overexpressed genes in *ABCG2*-high TCGA-STAD gastric cancer samples revealed significant enrichment of gastric cancer-associated oncogenic pathways. These include MAPK, PI3K-AKT, and ERBB signaling pathways, as well as pathways related to central carbon metabolism [47,48] (see Supplementary Figure S7). Activation of the MAPK/ERK signaling cascade plays a common role in gastric cancer progression, invasion, metastasis, and chemotherapy resistance. This highlights the clinical significance of the ERK pathway-related transcriptional programs observed in this study [47].

Our study suggests a functional relationship between *ABCG2* expression and ERK-dependent multidrug-resistant phenotypes in PTX-resistant gastric cancer cells. Although ERK inhibition and rescue experiments revealed an interaction between *ABCG2* and ERK signaling, the molecular mechanism linking *ABCG2* activity to ERK activation remains unclear. This interaction may involve receptor-associated signaling networks, intracellular stress responses, or indirect regulatory mechanisms. The present study was performed exclusively using in vitro gastric cancer models. Therefore, further in vivo validation using xenograft systems and patient-derived datasets will be necessary to confirm the biological and clinical relevance of the *ABCG2*–ERK signaling relationship.

From a therapeutic perspective, these findings suggest that targeting the *ABCG2*-ERK interaction could be a promising strategy to restore paclitaxel sensitivity in resistant gastric cancer cells. Pharmacological inhibition of ERK signaling reduced *ABCG2* expression and reversed multiple resistance-associated phenotypes. This supports the potential of pathway-focused combination therapies to overcome chemoresistance. Although this study used gastric cancer cell models, *ABCG2*-mediated multidrug resistance and ERK-associated signaling pathways have also been reported in many other tumor types [49,50]. Similar *ABCG2*- and ERK-associated resistance mechanisms may also exist in other tumor types. However, further research involving additional cancer models is needed to confirm this.

In conclusion, *ABCG2* was found to be related to multiple functions, including drug efflux activity, spheroid formation, and invasive capacity of gastric cancer cells with multidrug resistance. Our findings suggest a functional relationship between ERK signaling and the multidrug resistance and aggressive phenotypes associated with *ABCG2* in PTX-resistant gastric cancer cells.

Future studies should investigate the in vivo relevance of the association between *ABCG2* expression and ERK signaling using animal models. Evaluation of its clinical significance in patient-derived samples is crucial. In addition, identifying the regulatory mechanisms linking PTX exposure to *ABCG2* activation may contribute to the development of more effective therapeutic strategies to overcome multidrug resistance in gastric cancer.

## 4. Materials and Methods

### 4.1. Cell Culture and Establishment of PTX-Resistant Cells

HGC27 cell line (Accession CVCL_1279), derived from the metastatic lymph node of gastric cancer, and MKN45 cell line (Accession CVCL_0434), derived from the liver metastasis of a poorly differentiated gastric adenocarcinoma, were obtained from the Yeditepe University Cell Bank (Istanbul, Türkiye). Cells were cultured in RPMI medium (Diagnovum GmbH, Greifswald, Germany) supplemented with 10% fetal bovine serum (FBS; (Diagnovum GmbH, Greifswald, Germany) and antibiotics (100 U/mL penicillin and 100 µg/mL streptomycin). Cultures were maintained at 37 °C in a humidified incubator under a 5% CO2 atmosphere.

Resistant HGC27 and MKN45 cells (designated R HGC27 and R MKN45) were generated by gradually exposing the parental line to increasing concentrations of PTX (MedChemExpress, Monmouth Junction, NJ, USA). Selection began at 1/50 of the IC_50_ value, and the drug concentration was raised by 25% every two weeks. Cells were considered resistant once they could proliferate exponentially at PTX concentrations equivalent to the IC_50_ of their respective parental lines. The established resistant cells were maintained under identical culture conditions to their non-resistant counterparts, with the addition of PTX at its IC50 value [51].

### 4.2. Cell Viability Assay (MTT)

Cell viability and cytotoxicity were analyzed through the MTT (3-[4,5-dimethylthiazol-2-yl]-2,5 diphenyl tetrazolium bromide) assay (Sigma-Aldrich, St. Louis, MO, USA). It measures mitochondrial metabolic activity as an indicator of cell viability. Briefly, cells were seeded into 96-well plates at a density of 2 × 10^3^ cells per well in 100 µL of complete medium, and allowed to attach overnight under standard culture conditions. Each experimental condition was performed with at least five biological replicates.

The cells were exposed to increasing concentrations of PTX, cisplatin or 5-fluorouracil (5-FU). The control cells received 0.01% dimethyl sulfoxide (DMSO) as the vehicle control. After 48 h of treatment, 20 µL of MTT reagent and 80 µL of fresh culture medium were added to each well. The cells were then incubated at 37 °C for 2–4 h until visible formazan crystals formed. The culture medium was then carefully removed, and the resulting formazan crystals were dissolved in 100 µL of DMSO prior to measuring the absorbance.

Absorbance was measured at 570 nm using an Agilent BioTek Synergy H1 microplate reader (Agilent Technologies, Santa Clara, CA, USA). Cell viability was expressed as a percentage relative to the untreated control cells. The half-maximal inhibitory concentration (IC_50_) is defined as the concentration required to reduce cell viability by 50%. It was determined using linear regression analysis from three independent experiments. Each experiment was performed with at least five replicates.

### 4.3. Establishment of a Stable Cell Line

*ABCG2* was downregulated using an RNA interference-based approach. A lentiviral shRNA vector for *ABCG2* (sc-41151-V, Santa Cruz, CA, USA), and an appropriate empty control vector (sc-108080, Santa Cruz, CA, USA) were used. Cells were transduced with lentiviral particles according to the manufacturer’s instructions. Empty vector-transduced control cells, called sh-scramble, and sh-*ABCG2* stable cell lines were selected after treatment with 2 µg/mL puromycin for one month to establish stable knockdown cell lines. The silencing of *ABCG2* was confirmed through qRT-PCR and flow cytometry [52]. 

For ERK reactivation experiments, *ABCG2*-depleted cells were transduced with lentiviral particles encoding a constitutively active MEK1 mutant (caMEK1; Addgene plasmid, 64604, Watertown, MA, USA). Transduced cells were maintained under puromycin selection. ERK reactivation was confirmed by increased pERK/ERK levels and elevated expression of ERK downstream target genes.

### 4.4. ERK Pathway Inhibition

For inhibition of the ERK pathway, cells were treated with the ERK1/2 inhibitor PD98059 (Sigma-Aldrich, Darmstadt, Germany) at a final concentration of 20 µM for 24 h prior to downstream analyses [53].

### 4.5. Spheroid Formation Assay

Tumor spheroids were generated by seeding 4 × 10^4^ cells per well into 24-well plates that had been precoated with 2% agarose to prevent cell attachment. The HGC27 cells were cultured in a serum-free RPMI-1640 medium supplemented with 100 U/mL penicillin and 100 µg/mL streptomycin (Thermo Fisher Scientific, Waltham, MA, USA), without additional growth factors. After 10 days of incubation, spheroid formation was evaluated, and spheroid number was quantified using an inverted microscope (Leica Microsystems, Wetzlar, Germany) at 100× magnification [54].

### 4.6. In Vitro Cell Invasion Assay

The capacity of cell invasion was assessed using Matrigel-coated Transwell inserts (NEST Biotechnology Co., Ltd., Wuxi, Jiangsu, China). Briefly, 100 µL of Matrigel was coated onto the upper surfaces of the inserts, and the inserts were incubated at 37 °C for 30 min to allow polymerization of the gel. Then, a total of 4 × 10^4^ cells suspended in 200 µL of serum-free medium were seeded into the upper chambers. The lower chambers were then filled with 750 µL of RPMI-1640 medium containing 10% fetal bovine serum (FBS) to serve as a chemoattractant. After 24 h of incubation, the non-invading cells were removed and the membranes were fixed with 3.7% formaldehyde for two minutes, followed by permeabilization with absolute methanol for 20 min. The invaded cells were then stained with crystal violet for 15 min. Non-invading cells on the upper surface of the membranes were carefully removed with a cotton swab. In contrast, cells that had invaded the lower surface were visualized and quantified in four randomly selected fields per membrane using an inverted microscope at 400× magnification [55].

### 4.7. RNA Extraction and Quantitative Real-Time Polymerase Chain Reactions (qRT-PCR)

Cells were plated in T25 culture flasks at a density of 5 × 10^5^ cells per well and cultured for 48 h. Following incubation, cells were detached using trypsin, collected, and centrifuged at 400× *g* for 5 min. The resulting cell pellets were stored at −80 °C until further processing.

Total RNA was extracted using the innuPREP RNA Mini Kit 2.0 (IST Innuscreen GmbH, Berlin, Germany) according to the manufacturer’s protocol. Complementary DNA (cDNA) synthesis was carried out using the Wonder RT-cDNA Kit (Euroclone, Pero, Italy). The expression levels of 11 genes associated with cancer progression (listed in Table 1) were analyzed by quantitative real-time PCR using the FluoCycle II SYBR Master Mix Kit (Euroclone, Pero, Italy). *GAPDH* served as the internal reference gene, and relative mRNA expression levels were calculated using the Livak (2^−ΔΔC^_T_) method [56].

### 4.8. Annexin V-FITC/PI Apoptosis Assay

Apoptotic cell death was evaluated using the Annexin V-FITC/PI Apoptosis Kit (BioLegend, San Diego, CA, USA) in accordance with the manufacturer’s instructions. Briefly, 8 × 10^5^ cells were seeded into 6-well culture plates. After 24 h, cells were treated with PTX at IC_50_ concentrations appropriate for each experimental group.

Following 48 h of treatment, culture supernatants were collected into centrifuge tubes, and adherent cells were gently washed with phosphate-buffered saline (PBS); the wash solutions were combined with the corresponding supernatants. Cells were then detached by trypsinization, pooled with the collected fractions, and centrifuged at 300× *g* for 5 min. The resulting pellets were washed twice with ice-cold PBS, with centrifugation at 300× *g* for 5 min after each wash.

Cell pellets were resuspended in 500 µL of 1× Annexin V Binding Buffer, followed by the addition of 5 µL Annexin V-FITC and 5 µL propidium iodide (PI). Samples were gently mixed and incubated for 20 min at room temperature in the dark. Apoptotic populations were analyzed within 1 h using a CytoFLEX flow cytometer (Beckman Coulter, Brea, CA, USA).

### 4.9. Cell Cycle Analysis

Cells were collected and washed with phosphate-buffered saline (PBS). Fixation was performed by gradually adding ice-cold ethanol to the cell suspension while gently vortexing, resulting in a final ethanol concentration of 70%. The cells were kept at 4 °C for at least 2 h. After fixation, the cells were washed once with PBS and resuspended in a staining buffer consisting of PBS containing 100 μg/mL RNase A and 50 μg/mL propidium iodide (PI). To improve membrane permeabilization, 0.1% Triton X-100 was added to the staining solution. All steps involving the PI were conducted in the absence of light. The cells were incubated in the staining buffer overnight at 4 °C, and DNA content was subsequently analyzed using a CytoFLEX flow cytometer (Beckman Coulter, Brea, CA, USA).

### 4.10. Efflux Pump Assay

A total of 1 × 10^5^ cells/well were seeded into black-walled, clear-bottom 96-well plates (Sigma-Aldrich) and allowed to adhere overnight. Once the cells reached approximately 70% confluence, the culture medium was removed, and the cells were washed twice with glucose-supplemented PBS(+), consisting of 0.9 mM CaCl_2_·2H_2_O, 0.33 mM MgCl_2_·12H_2_O, and 10 mM glucose. Subsequently, 100 µL of PBS(+) glucose buffer was added to each well, and the plates were incubated for 1 h at 37 °C.

After incubation, the buffer was replaced with 100 µL of Calcein-AM solution (4 μM) prepared in RPMI-1640 medium, and the cells were incubated for an additional 1 h at 37 °C. The staining solution was then aspirated, and the cells were washed with ice-cold PBS(+). Cell lysis was achieved by adding 100 µL of 1% SDS in PBS and incubating the plates for 10 min at room temperature in the dark. Intracellular calcein fluorescence, observed as a strong yellow–green signal, was measured using Synergy H1 Microplate Reader (BioTek Instruments, Winooski, VT, USA). Data are expressed as mean ± standard deviation from at least three independent experiments [27].

### 4.11. Flow Cytometry Analysis

Cells were washed once with PBS, detached using a cell scraper (NEST Biotechnology Co., Ltd., Wuxi, Jiangsu, China), and centrifuged to collect cell pellets. The cells were then resuspended and incubated with a CD44-specific antibody (Catalog no. 555478, BD Pharmingen, San Jose, CA, USA) and an LGR5-specific antibody (Catalog no. 373803, BioLegend, San Diego, CA, USA) and incubated with antibodies for 30 min at 4 °C in the dark. Flow cytometric analysis was performed using a CytoFLEX flow cytometer (Beckman Coulter, Brea, CA, USA). Instrument settings and analysis parameters were established based on unstained control samples.

### 4.12. Determination of Protein Levels by ELISA

Cells (5 × 10^6^) were plated in T75 culture flasks and incubated for 24 h to allow attachment. Subsequently, the medium was replaced with RPMI-1640 alone as the untreated control or RPMI-1640 containing the IC_50_ dose of PTX. Total cellular proteins were extracted using standard lysis procedures, and protein concentrations were quantified using the bicinchoninic acid (BCA) assay. Protein expression levels were subsequently evaluated using commercially available assay kits. Total and phosphorylated ERK1/ERK2 levels were measured using the ERK1/ERK2 (Total/Phospho) Multispecies InstantOne ELISA Kit (Catalog no. 85-86013-11; Thermo Fisher Scientific, Waltham, MA, USA) in accordance with the manufacturer’s protocol [57].

### 4.13. Statistical Analysis

Statistical analyses were conducted using the Kruskal–Wallis test, followed by the Mann–Whitney U test for pairwise comparisons. A *p*-value of <0.05 was considered to indicate statistical significance and is denoted by an asterisk (* or #). All experiments were performed in at least five independent biological replicates.

## Figures and Tables

**Table 1:** The primer sequences for qRT-PCR.

Gene	Primers
*GAPDH*	Forward: 5′-GCACCGTCAAGGCTGAGAAC-3′ Reverse: 5′-ATGGTGGTGAAGACGCCAGT-3′
*OCT4 (POU5F1)*	Forward: 5′-CCTGAAGCAGAAGAGGATCACC-3′ Reverse: 5′-AAAGCGGCAGATGGTCGTTTGG-3′
*NANOG*	Forward: 5′-CTCCAACATCCTGAACCTCAGC-3′ Reverse: 5′-CGTCACACCATTGCTATTCTTCG-3′
*SNAIL (SNAI1)*	Forward: 5′-GCACATCCGAAGCCACACGC-3′ Reverse: 5′-CTTGACATCTGAGTGGGTCTGG-3′
*TWIST (TWIST1)*	Forward: 5′-CTGGCGGCCAGGTACATCGAC-3′ Reverse: 5′-GGACGCGGACATGGACCAGGCC-3′
*MMP2*	Forward: 5′-AGCGAGTGGATGCCGCCTTTAA-3′ Reverse: 5′-CATTCCAGGCATCTGCGATGAG-3′
*ERCC1*	Forward: 5′-CTGGGAATTTGGCGACGTAA-3′ Reverse: 5′-ATGGATGTAGTCTGGGTGCAG-3′
*SOX2*	Forward: 5′-GCTACAGCATGATGCAGGACCA-3′ Reverse: 5′-TCTGCGAGCTGGTCATGGAGTT-3′
*SOX9*	Forward: 5′-AGGAAGCTCGCGGACCAGTAC-3′ Reverse: 5′-GGTGGTCCTTCTTGTGCTGCAC-3′
*N-CADHERIN (CDH2)*	Forward: 5′-CCTCCAGAGTTACTGCCATGAC-3′ Reverse: 5′-GTAGGATCTCCGCCACTGATTC-3′
*E-CADHERIN (CDH1)*	Forward: 5′-GCCTCCTGAAAAGAGAGTGGAAG-3′ Reverse: 5′-TGGCAGTGTCTCTCCAAATCCG-3′
*ABCG2*	Forward: 5′-GTTCTCAGCAGCTCTTCGGCTT-3′ Reverse: 5′-TCCTCCAGACACACCACGGATA-3′
*JUN*	Forward: 5′-CCTTGAAAGCTCAGAACTCGGAG-3′ Reverse: 5′-TGCTGCGTTAGCATGAGTTGGC-3′
*EGR1*	Forward: 5′-AGCAGCACCTTCAACCCTCAGG-3′ Reverse: 5′-GAGTGGTTTGGCTGGGGTAACT-3′
*ETV4*	Forward: 5′-AGGAACAGACGGACTTCGCCTA-3′ Reverse: 5′-CTGGGAATGGTCGCAGAGGTTT-3′

## Data Availability

The original contributions presented in this study are included in the article/Supplementary Material. Further inquiries can be directed to the corresponding author.

## Supplementary Materials

The following supporting information can be downloaded at: https://www.mdpi.com/article/10.3390/ijms27115039/s1.

## Abbreviations

The following abbreviations are used in this manuscript:

PTX	Paclitaxel
CSCs	Cancer Stem Cells
MDR	Multidrug Resistance
PBS	Phosphate-Buffered Saline
EMT	Epithelial–Mesenchymal Transition
*ABCG2*	ATP-Binding Cassette Subfamily G Member 2
ERK	Extracellular Signal-Regulated Kinase
MAPK	Mitogen-Activated Protein Kinase
qRT-PCR	Quantitative Real-Time Polymerase Chain Reaction
FITC	Fluorescein Isothiocyanate
PE	Phycoerythrin
RFU	Relative Fluorescence Unit
IC50	Half-Maximal Inhibitory Concentration
caMEK1	Constitutively Active MEK1
5-FU	5-Fluorouracil
FBS	Fetal Bovine Serum
SD	Standard Deviation
RT-PCR	Reverse Transcription Polymerase Chain Reaction
TCGA	The Cancer Genome Atlas
STAD	Stomach Adenocarcinoma
RPPA	Reverse-Phase Protein Array
KEGG	Kyoto Encyclopedia of Genes and Genomes
