# Supplementary material for: ABCG2 Contributes to Multidrug Resistance and Aggressive Phenotypes Associated with ERK Signaling in Gastric Cancer
Source: Int J Mol Sci. 2026 Jun 2;27(11):5039. doi: 10.3390/ijms27115039 (PMC13256579; doi:10.3390/ijms27115039)
Supplement: Supplementary file 1 [file ijms-27-05039-s001.zip › ijms-4247959-supplementary-updated on 6.8.pdf]

Supplementary Material

Supplementary Methods:

Bioinformatic analysis using the TCGA-STAD dataset

Publicly available transcriptomic and clinical data from The Cancer Genome Atlas stomach adenocarcinoma (TCGA-STAD) cohort were used to explore the clinical relevance of the experimental findings. Gene expression correlation analyses were performed using the cBioPortal for Cancer Genomics platform. The Stomach Adenocarcinoma (TCGA, PanCancer Atlas) dataset was queried, and the mRNA expression z-scores relative to the log RNA-Seq V2 RSEM profile of all samples were selected. Only cases with available mRNA expression data were included in the analysis. Pairwise correlations between ABCG2 and selected genes associated with the ERK pathway and stemness were evaluated using the platform-provided correlation coefficients (Spearman's and Pearson's). Correlations with $p < 0.05$ were considered statistically significant.

An additional gene expression correlation analysis was performed using UCSC Xena browser on the TCGA Stomach Adenocarcinoma (TCGA-STAD) cohort. Gene expression data were obtained from the TCGA HiSeq RNA-seq dataset and expressed as log2(norm_count+1) values. Associations between gene expression levels were evaluated using Pearson's and Spearman's rank correlation analyses. An overall survival analysis based on ABCG2 expression in gastric cancer patients was performed using the Kaplan–Meier Plotter database (https://kmplot.com/analysis/), which combines gene expression and survival data from several public gastric cancer cohorts. Patients were stratified into high- and low-expression groups using the 'auto-selected best cutoff' option. The platform automatically calculated hazard ratios (HRs) with 95% confidence intervals and log-rank p-values.

An additional Kaplan–Meier survival analysis was performed using the UCSC Xena browser (https://xenabrowser.net/) based on the TCGA stomach cancer (STAD) cohort. ABCG2 expression data generated by Illumina HiSeq RNA-seq were used for survival stratification. Patients were stratified by ABCG2 expression quartiles, and overall survival was analyzed by comparing the highest and lowest quartiles. Statistical significance was evaluated using the log-rank test, and the corresponding p-value was recorded from the platform output.

UALCAN/CPTAC Proteomic Analysis

The proteomic expression profiles of MAPK1 and MAPK3 in gastric cancer were analyzed using the UALCAN platform, based on the CPTAC gastric cancer cohort. The protein expression levels across pan-cancer proteome-based subtypes were compared using normalized Z-values derived from CPTAC mass spectrometry datasets. Statistical comparisons were performed using the UALCAN platform.

Correlation Analysis of ABCG2 Expression and ERK-Related Phosphoprotein Signaling

Correlation analyses were performed using the cBioPortal TCGA stomach adenocarcinoma (STAD) dataset. Associations between ABCG2 mRNA expression and RPPA-based phosphoprotein expression levels were evaluated using Spearman's correlation analysis. ERK downstream signaling components, including phosphorylated JUN (JUN_pS73) and phosphorylated RPS6KA1 (RPS6KA1_pT359/S363), were analyzed to assess the potential association between ABCG2 expression and ERK-related signaling activity in gastric cancer.

KEGG Pathway Enrichment Analysis

Using cBioPortal, genes that were overexpressed in ABCG2-high gastric cancer samples from the TCGA-STAD cohort (mRNA z-score >2) were identified. A focused gene set associated with RTK/MAPK signaling, proliferation, and aggressive tumor phenotypes was subjected to Kyoto Encyclopedia of Genes and Genomes (KEGG) pathway enrichment analysis using the Enrichr platform (https://maayanlab.cloud/Enrichr/enrich , accessed on May 13, 2026).

Limiting dilution spheroid formation assay:

Cells were dissociated into single-cell suspensions and seeded into agarose-coated 96-well plates at limiting dilutions of 1 cell per well in serum-free spheroid culture medium. Wells containing single cells were visually confirmed under an inverted microscope immediately after plating. Cells were cultured for 15 days, and the number of wells containing spheroids was counted. Sphere-forming frequency was calculated as the percentage of wells containing at least one spheroid relative to the total number of wells plated.

Immunofluorescence Analysis:

The cells were seeded onto sterile glass coverslips and left to attach overnight. After treatment, the cells were fixed with 4% paraformaldehyde for 15 minutes at room temperature, and permeabilized with 0.1% Triton X-100 for 10 minutes. After blocking with 3% bovine serum albumin (BSA) for 30 minutes, the cells were incubated overnight at 4 °C with a phospho-ERK1/2 (Thr202/Tyr204) monoclonal antibody (clone MILAN8R, eBioscience™, 14-9109-82, Thermo Fisher Scientific, Waltham, MA, USA). After washing with PBS, the cells were incubated with an Alexa Fluor 594-conjugated goat anti-mouse IgG secondary antibody (1:500 dilution) (A21125, Thermo Fisher Scientific, Waltham, MA, USA) for 1 hour at room temperature in the dark. The nuclei were counterstained with DAPI, and the coverslips were mounted using an antifade mounting medium. Images were captured using identical exposure settings across all experimental groups to allow quantitative comparison. Immunofluorescence images were analyzed using ImageJ software. Nuclear regions were defined based on DAPI staining, and cytoplasmic regions were defined based on cell boundaries. The fluorescence intensity of pERK was measured in both compartments, and nuclear localization was quantified as the nuclear-to-cytoplasmic fluorescence intensity ratio. At least 20 cells per group were analyzed per experiment from representative images obtained in five independent experiments. Image acquisition parameters were kept constant across all experimental groups.

Supplementary Figures:

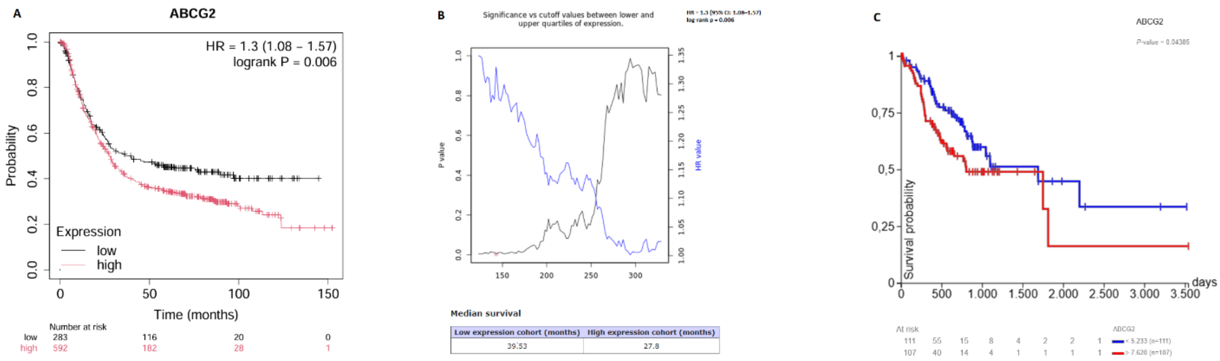

Supplementary Figure S1. Clinical relevance of ABCG2 expression in gastric cancer patients. A) Kaplan–Meier survival analysis using the KM-plotter gastric cancer dataset showed that high ABCG2 expression was significantly associated with reduced overall survival (HR = 1.3, 95% CI: 1.08–1.57, log-rank p = 0.006). B) Auto-cutoff plot showing the relationship between expression cutoff values and survival significance. C) Kaplan–Meier survival analysis using the TCGA-STAD RNAseq dataset accessed through the UCSC Xena platform confirmed the association between elevated ABCG2 expression and poorer overall survival (log-rank p = 0.04385). (https://kmplot.com/analysis/; accessed on April 16, 2026, https://xenabrowser.net/; accessed on April 16, 2026).

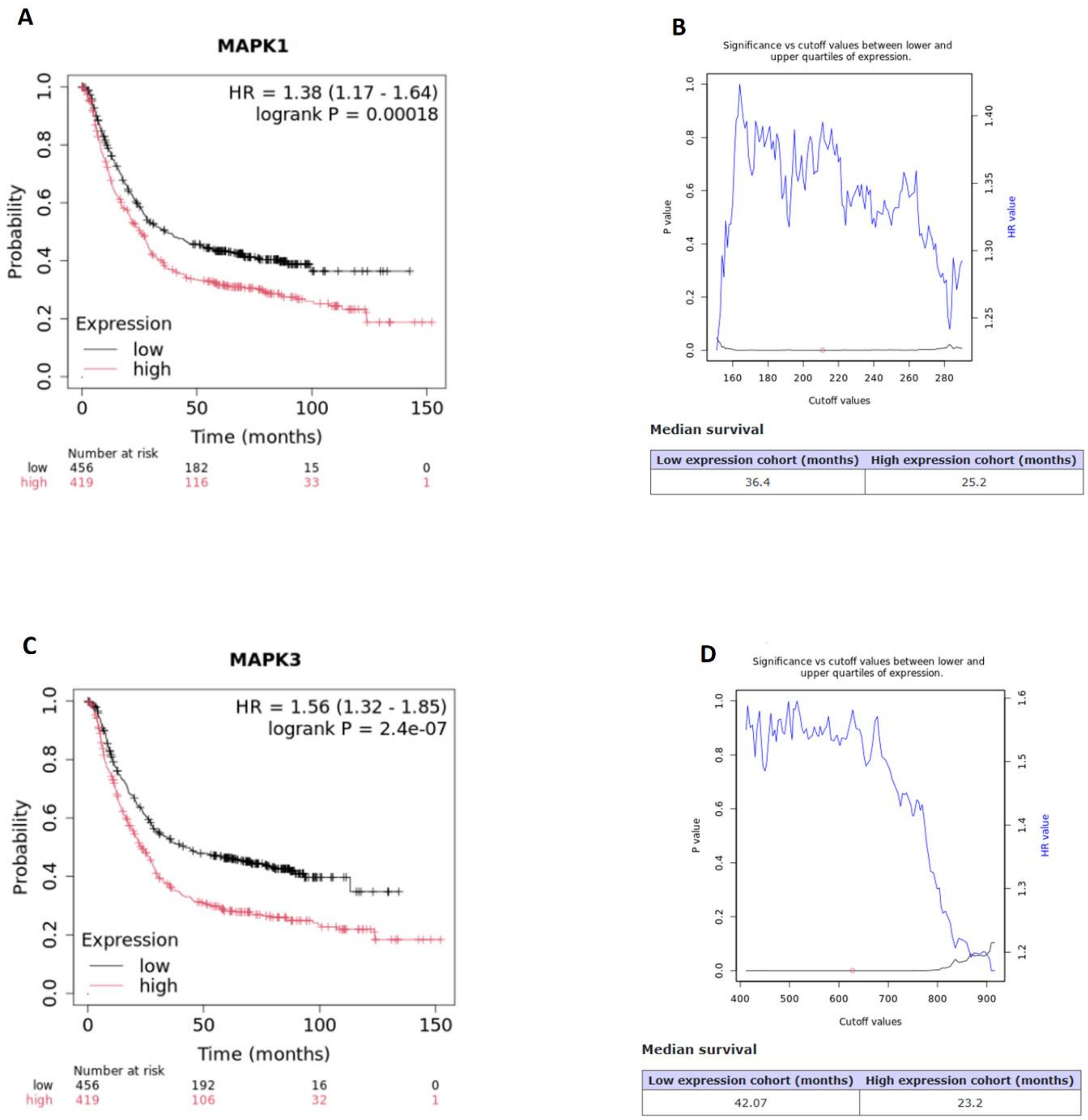

Supplementary Figure S2. Kaplan–Meier survival analysis of MAPK1 and MAPK3 expression in gastric cancer patients. A, B) Elevated MAPK1 expression was significantly associated with poorer overall survival (HR = 1.38, 95% CI: 1.17–1.64; log-rank p = 0.00018; FDR = 2%) based on analysis using the KM-plotter database. C, D) Elevated MAPK3 expression was significantly associated with poorer overall survival (HR = 1.56, 95% CI: 1.32–1.85; log-rank p = 2.4 × 10^{-7}; FDR = 1%) based on analysis using the KM-plotter database. (https://kmplot.com/analysis/; accessed on April 16, 2026)

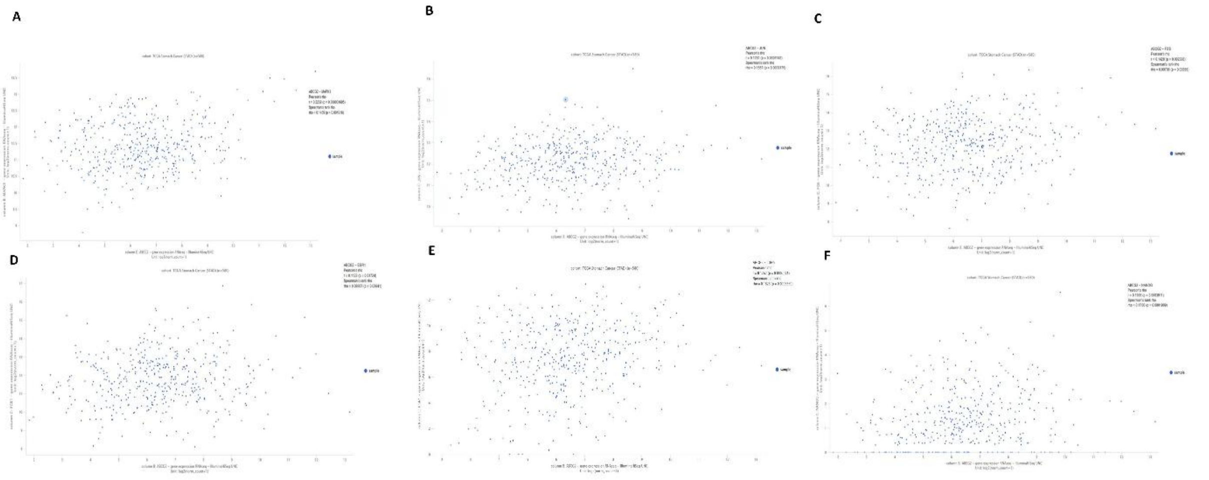

Supplementary Figure S3. **A correlation analysis was performed using the UCSC Xena browser to examine the relationship between ABCG2 expression and ERK pathway components (*MAPK3, JUN, FOS*, and *EGR1*), as well as stemness-associated markers (*LGR5* and *NANOG*), in gastric cancer patients from the TCGA-STAD cohort. Gene expression values were obtained from TCGA RNA-seq datasets and are presented as $\log_2$(norm_count+1)-transformed values. Pearson's and Spearman's correlation coefficients are shown.**

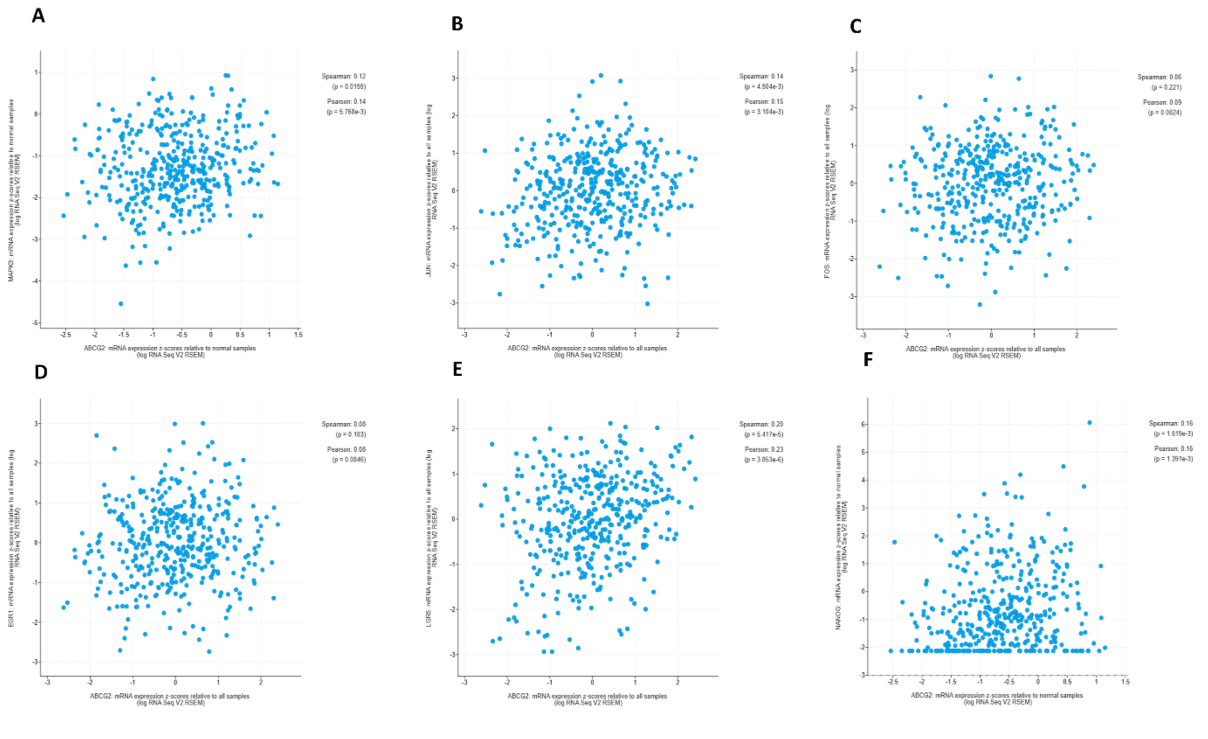

Supplementary Figure S4. **A correlation analysis was performed using the cBioPortal platform to examine the relationship between *ABCG2* expression, ERK signaling, and stemness-related genes (*MAPK3, JUN, FOS, EGR1, LGR5*, and *NANOG*) in gastric cancer patients from the TCGA-STAD cohort. Expression values were obtained from log-transformed RNA-Seq V2 RSEM data. The results show Pearson's and Spearman's correlation coefficients.**

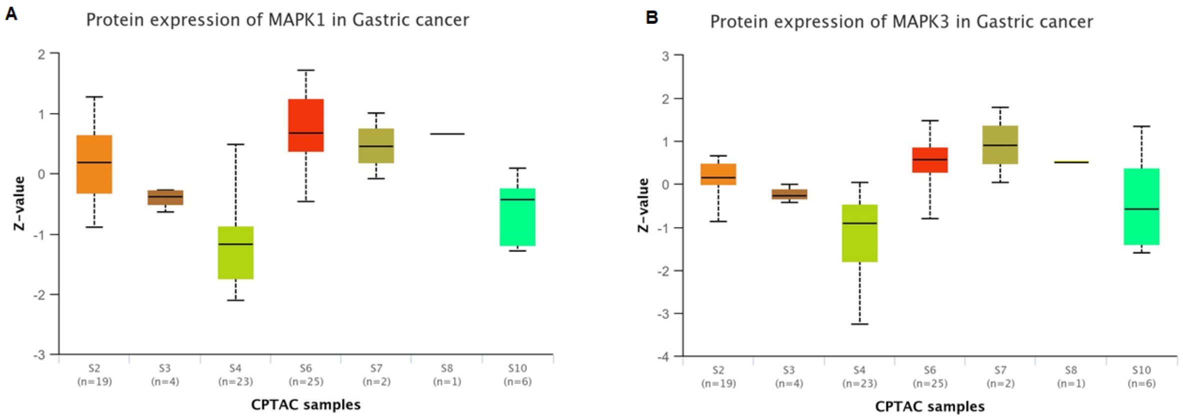

Supplementary Figure S5. CPTAC/UALCAN-based proteomic expression analysis of MAPK1 and MAPK3 in gastric cancer. Boxplots demonstrate subtype-dependent differences in A) MAPK1 (ERK2) and B) MAPK3 (ERK1) protein expression across pan-cancer proteomic subtypes of gastric cancer. Protein expression values are presented as normalized Z-scores obtained from CPTAC proteomic datasets. Pairwise statistical comparisons are provided by the UALCAN platform

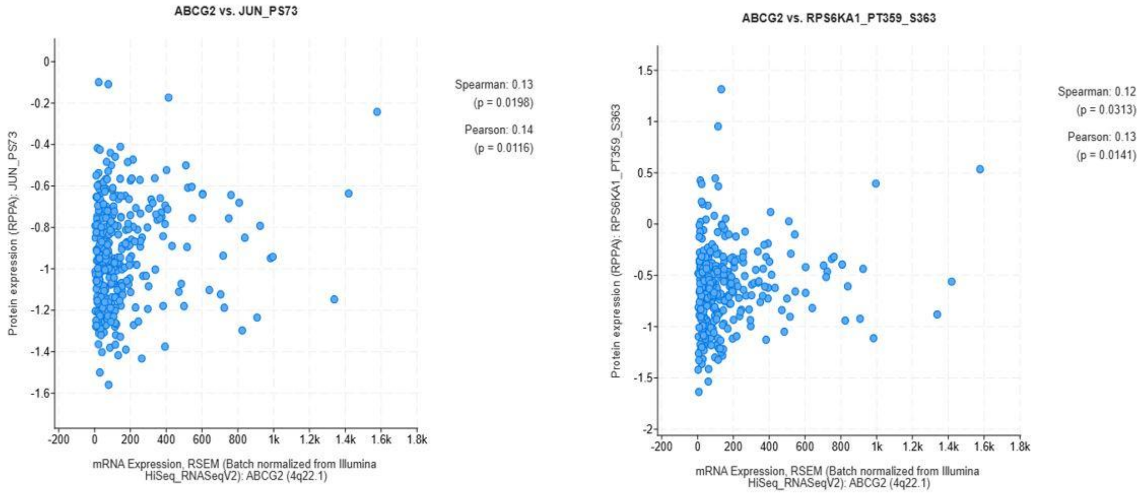

Supplementary Figure S6. Correlation analysis between ABCG2 expression and ERK downstream phosphoprotein markers in TCGA-STAD samples. RPPA-based analyses demonstrated modest but significant positive correlations between ABCG2 mRNA expression and phosphorylated JUN (JUN_pS73) as well as phosphorylated RPS6KA1 (RPS6KA1_pT359/S363). These findings support a potential association between ABCG2 expression and ERK-related downstream signaling activity in gastric cancer.

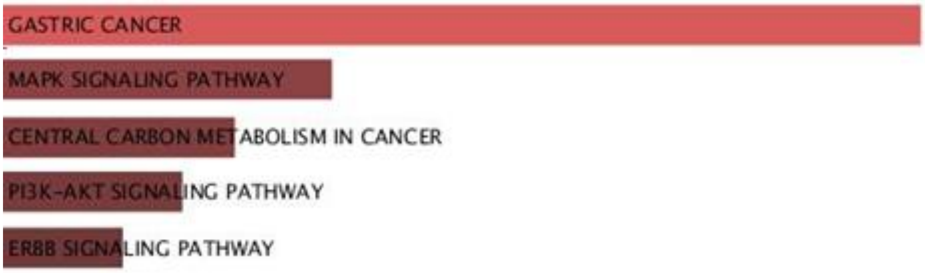

Supplementary Figure S7. An exploratory KEGG pathway enrichment analysis was performed on genes that were overexpressed in ABCG2-high gastric cancer cases from the TCGA-STAD cohort. The Enrichr platform was used to perform the enrichment analysis. Pathways associated with gastric cancer progression and oncogenic signaling, including the MAPK, PI3K-AKT, and ERBB signaling pathways, were significantly enriched.

Supplementary Table S1. Selected enriched KEGG pathways associated with recurrently overexpressed genes identified in ABCG2-high gastric cancer cases.

Pathway	Adjusted p-value	Odds ratio
Gastric cancer	0.0027	59.48
MAPK signaling pathway	0.0087	28.81
Central carbon metabolism in cancer	0.0087	75.47
PI3K-Akt signaling pathway	0.0087	23.91
ERBB signaling pathway	0.0091	61.45

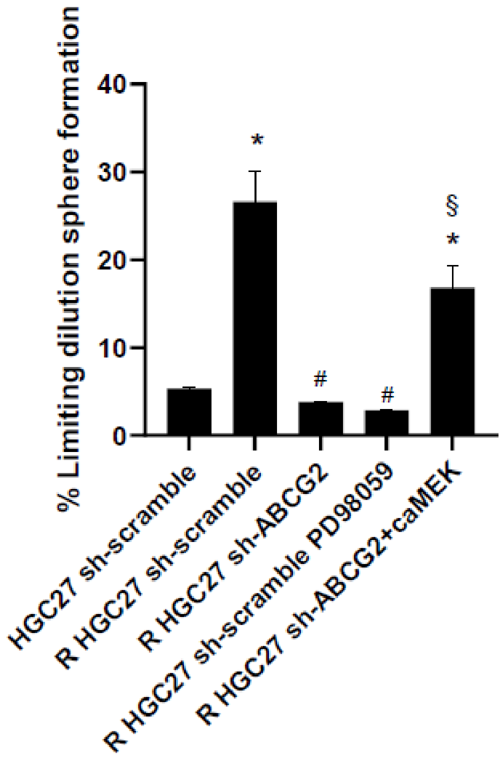

Supplementary Figure S8. Single-cell suspensions were seeded into agarose-coated 96-well plates at 1 cell/well and cultured under spheroid-forming conditions for 15 days. The percentage of spheroid-positive wells was calculated for each group. R HGC27 cells showed an increased frequency of sphere initiation compared with parental cells, whereas ABCG2 silencing or ERK inhibition (PD98059) reduced sphere formation capacity. Restoration of ERK signaling by a constitutively active MEK partially restored sphere-initiation capacity in ABCG2-silenced cells. Data are presented as mean ± SD. *p < 0.05 vs HGC27 sh-scramble; #p < 0.05 vs R HGC27 sh-scramble; §p < 0.05 vs R HGC27 sh-ABCG2

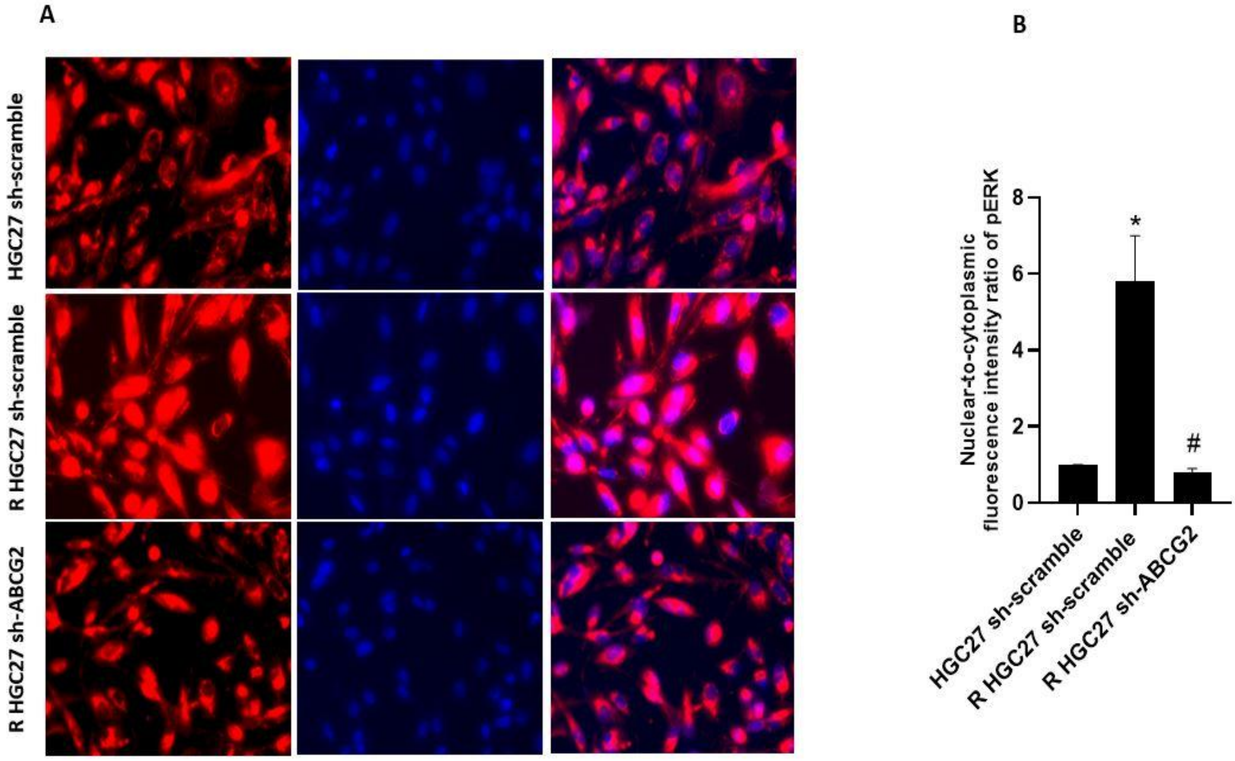

Supplementary Figure S9. Nuclear localization of phosphorylated ERK (pERK) in paclitaxel-resistant gastric cancer cells following ABCG2 silencing. A) Immunofluorescence staining of pERK (red) and nuclei (DAPI, blue) in HGC27 sh-scramble, R HGC27 sh-scramble, and R HGC27 sh-ABCG2 cells. Representative merged images show increased nuclear accumulation of pERK in resistant cells compared with parental cells, which was reduced following ABCG2 knockdown. B) Quantification of nuclear-to-cytoplasmic fluorescence intensity ratios of pERK measured using ImageJ. Images were acquired using identical exposure settings. Data are presented as mean ± SD. *p < 0.05 vs HGC27 sh-scramble; #p < 0.05 vs R HGC27 sh-scramble.

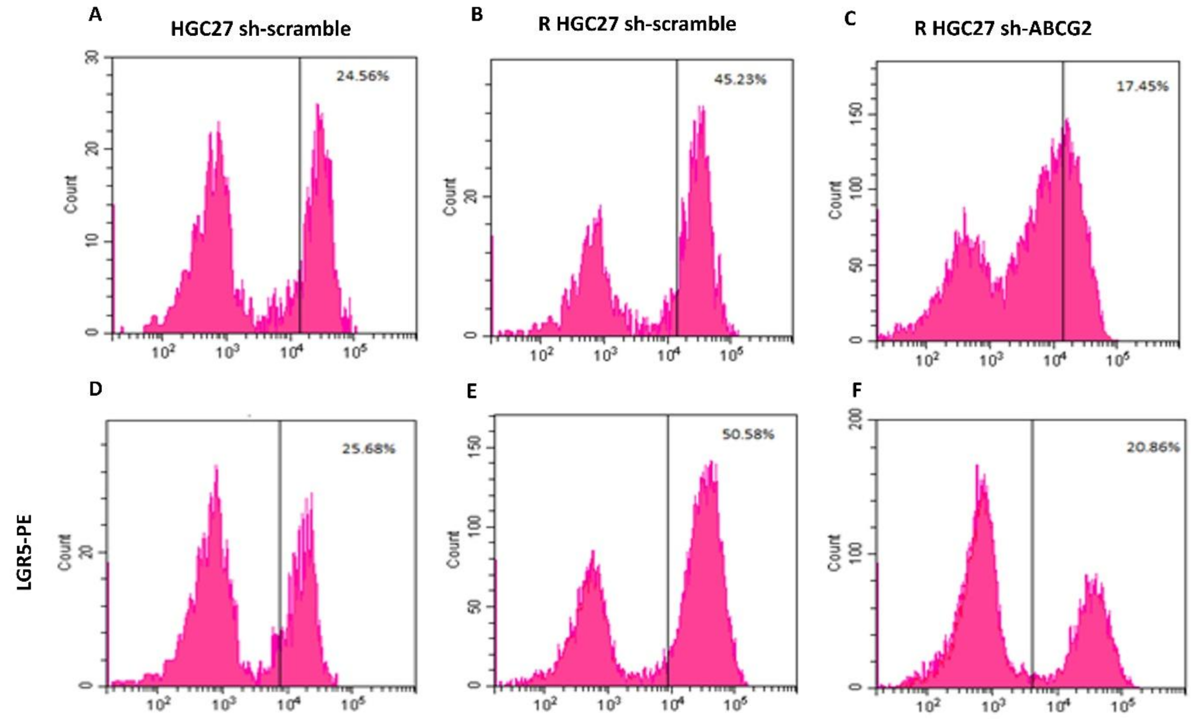

Supplementary Figure S10. Representative flow cytometry histograms showing CD44- and LGR5-positive cell populations in HGC27 and multidrug-resistant HGC27 cells. A–C) Representative flow cytometry histograms showing CD44-FITC expression in HGC27 sh-scramble, R HGC27 sh-scramble, and R HGC27 sh-ABCG2 cells. D–F) Representative flow cytometry histograms showing LGR5-PE expression in the indicated cell groups.
